# A prophage encoded ribosomal RNA methyltransferase regulates the virulence of Shiga-toxin-producing *Escherichia coli* (STEC)

**DOI:** 10.1093/nar/gkad1150

**Published:** 2023-12-12

**Authors:** Chen Gong, Dolonchapa Chakraborty, Gerald B Koudelka

**Affiliations:** Department of Biological Sciences University at Buffalo, Buffalo, NY 14260, USA; Department of Biological Sciences University at Buffalo, Buffalo, NY 14260, USA; Department of Biological Sciences University at Buffalo, Buffalo, NY 14260, USA

## Abstract

Shiga toxin (Stx) released by Shiga toxin producing *Escherichia coli* (STEC) causes life-threatening illness. Its production and release require induction of Stx-encoding prophage resident within the STEC genome. We identified two different STEC strains, PA2 and PA8, bearing Stx-encoding prophage whose sequences primarily differ by the position of an IS*629* insertion element, yet differ in their abilities to kill eukaryotic cells and whose prophages differ in their spontaneous induction frequencies. The IS*629* element in ϕPA2, disrupts an ORF predicted to encode a DNA adenine methyltransferase, whereas in ϕPA8, this element lies in an intergenic region. Introducing a plasmid expressing the methyltransferase gene product into ϕPA2 bearing-strains increases both the prophage spontaneous induction frequency and virulence to those exhibited by ϕPA8 bearing-strains. However, a plasmid bearing mutations predicted to disrupt the putative active site of the methyltransferase does not complement either of these defects. When complexed with a second protein, the methyltransferase holoenzyme preferentially uses 16S rRNA as a substrate. The second subunit is responsible for directing the preferential methylation of rRNA. Together these findings reveal a previously unrecognized role for rRNA methylation in regulating induction of Stx-encoding prophage.

## Graphical Abstract

**Graphical Abstract:**
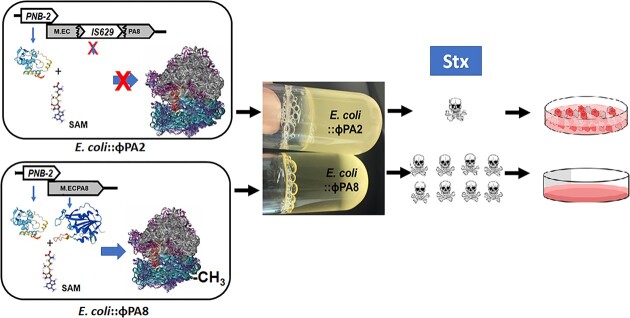

## Introduction

Shiga toxin producing *Escherichia coli* (STEC) are foodborne pathogens of emerging importance. STEC infections present a significant public health challenge, annually causing 265000 illnesses that result in economic impacts exceeding $405 million in the United States alone (1–3). Depending on the precise STEC strain, between 5 and 20% of patients with STEC symptoms develop hemolytic uremic syndrome (HUS) (4–6), which has a 3–5% mortality rate. Also, between 5–10% of infected individuals are left with permanent renal or neural sequelae (7–9). Given the health and economic impacts of STEC infections, it is essential that effective prevention and/or treatment regimens be developed.

Shiga toxin (Stx) is the essential virulence factor of STEC. Stx is encoded on a temperate bacteriophage and all STEC strains harbor at least one Stx-encoding prophage. Although *E. coli* serotype O157:H7 is a leading cause of STEC infections in humans, a large number of STEC serotypes cause human disease (10). Importantly, STEC outbreaks are increasingly caused by these non-O157 strains (11); the proportion of non-O157 STEC infections has increased recently to 64% in United States and 68.1% worldwide (12–14). There are two major antigenic forms of Stx, Stx1 and Stx2 (15). While many STEC strains encode more than one antigenic form, epidemiological studies suggest that Stx2 is more often associated with severe disease and development of HUS than Stx1 (16,17).

All Stx isoforms are ribosome inactivating toxins. Stx kills cells by removing an adenine from the sarcin-ricin loop of the large subunit rRNA, consequently inhibiting protein synthesis (18). The sequence of this loop is highly conserved between eukaryotes and bacteria (19,20), thus the ribosomes of both are vulnerable to attack by Stx (21,22).

STEC seemingly arose when Stx-encoding λ-like converting bacteriophage(s) infected benign or mildly pathogenic *E. coli* strains. Upon infecting a cell, all λ-like phages choose between two developmental fates, lysogeny or lysis. In lysogenic growth, the phage chromosome inserts into the host genome and the gene regulatory activities of the phage-encoded DNA binding *c*I repressor protein allow the prophage to lay essentially dormant. Removal of the *c*I protein from DNA allows the prophage switch to lytic growth (23–25). During lytic growth the prophage excises itself from the host genome, replicates and packages its DNA and lyses the host. Since the promoters governing transcription of the Stx encoding genes are only highly active during lytic growth, and Stx protein cannot be transported out the cell, high level Stx production and its subsequent release only occurs during phage lytic growth (26–29). Thus, prophage induction and subsequent lysis of the bacterial host are essential to STEC pathogenesis.

Lambdoid prophages can either spontaneously switch from lysogeny to lytic growth, or this process can be triggered by agents that activate the host DNA damage response (SOS) pathway (30,31). Stx prophage lysogens contain lower intracellular levels of their *c*I repressor than do bacteria harboring non-Stx encoding lambdoid phage (32,33). As such in Stx prophage lysogens, less repressor is available to occupy its binding sites. This condition contributes to the much higher rates of spontaneous induction exhibited by Stx-encoding prophage, in comparison to other lambdoid prophages (34,35). Importantly, high virulence strains show much higher levels of spontaneous Stx prophage induction, and it is thought that high spontaneous induction rates contribute to the development of HUS in the patients receiving non-antibiotic therapies (35–38). In damage response triggered (SOS)—prophage induction, activated RecA stimulates the autocleavage of *c*I repressor (39,40). This cleavage dramatically lowers the repressor's affinity for its DNA binding sites. Hence, both spontaneous and SOS-mediated prophage induction mechanisms involve the loss/lower occupancy of *c*I repressor binding sites in the phage genome.

Many commonly used antibiotics induce the SOS response (e.g. quinolone antibiotics), thereby inducing lytic growth of Stx-encoding prophage (26) and causing increased production and release of Stx (30,31). STEC-infected patients, especially children, treated with certain antibiotics have a much higher chance of developing HUS (9,41,42). Thus, treatment of STEC infections with antibiotics is contraindicated. While it is clear that transcriptional and translational inhibitors can be used to inhibit Stx production (43) *in vitro* several studies suggest that administration of any antibiotic increases the risk of severe STEC-mediated disease (44,45). Consequently, WHO and CDC recommend against treating STEC infections with antibiotics (46,47). Accordingly, current management of STEC infections relies mainly on supportive therapies, although causative therapies may be available (41,42).

Interestingly, not all STEC strains are equally capable of causing pathogenesis in humans. The cause of the variation in virulence between strains is unclear. Several observations suggest that STEC virulence differences are governed, at least in part, by the characteristics of the particular Stx-encoding prophage in a given strain. Supporting this suggestion are the observations that (i) the Stx-encoding prophage population varies between outbreaks with different severity and these prophages are divergent while the genomic structure of their *E. coli* hosts are more homogenous and (ii) prophage-encoded factors modify STEC gene expression and consequently its virulence-associated phenotypes (48–51). Additionally, several studies indicate that prophage genes regulate host behavior, yet it is unclear whether these phage-encoded factors modulate the host machinery that regulates prophage induction (52–55).

Here, we show that a prophage-encoded methyltransferase, apparently uniquely encoded by Stx-encoding phages and common among them, regulates prophage induction and affects STEC virulence. We found that the methyltransferase protein forms a heteromer with another protein that shows structural similarity to nucleic acid binding proteins and that this heteromeric methyltransferase holoenzyme preferentially uses 16S rRNA of host *E. coli* as its substrate. These findings suggest that rRNA methylation plays a heretofore unrecognized role in regulating prophage induction and virulence.

## Methods and materials

***Strains*** *Acanthamoeba castellanii* strain (*A*. *castellanii* ATCC 30234) and *E coli*. MG1655 strain were obtained from the American Type Culture Collection (Manassas, VA). *E*. *coli* O157:H7 strains containing φPA2 or φPA8 were a generous gift from Dr. Edward G Dudley, Penn State University. Each strain harbors only one Stx-encoding prophage and those prophages encode *stx*2a. The genomes of these strains and the isolated phages have been sequenced (GenBank accession numbers: *Escherichia coli* PA2 NZ_CP072564; *Escherichia coli* PA8 NZ_CP072565; ϕPA2 KP682371.1; ϕPA8 KP682374 (56). *E. coli* strain GM48 (*dcm^−^ dam^−^*) was obtained from the Coli Genetic Stock Center (Yale University) and RW102 (a *dam^−^* derivative of JM101) was a gift from Robin Wharton (Ohio State University).

PA2 and PA8 phages were obtained by inducing *E. coli* PA2 or *E. coli* PA8 strains with 50 μg/ml ciprofloxacin for 8h. Respective lysogens in MG1655 were constructed as described (57,58). Briefly, dilutions of phages isolated from the parental EHEC strains were spotted on a lawn of MG1655 growing in soft agar overlaid on an LB agar plate and the plates incubated overnight at 37°C to allow the formation of phage plaques. Putative lysogens were picked from the center of these plaques and streaked on an LB agar plate. Successful lysogenization was verified by colony PCR.

***Plasmid construction*** The p-M.EcoPA8 plasmid constitutively expressing the phage-encoded putative methyltransferase (M.EcoPA8orf6770P) was ordered from Genewiz (South Plainfield, NJ). For simplicity, since this ORF is the 5th gene from the left end of the phage genome, by convention, we will refer to the gene encoding M.EcoPA8orf6770P as *gp*5. The *gp*5 sequence in p-M.EcoPA8 is fused to the sequence encoding a 6 amino acid His-tag at its 5′ end inserted into the *Eco*RV site of pUC57(KAN). Primers used in this study were obtained from Genewiz (South Plainfield, NJ). The p-M.EcoPA8 plasmid directing expression of a mutant enzyme was created by site-directed mutagenesis using primers to modify the conserved SAM binding sites DPPW to APPA (5′-CACTCTCATTTACGCAGCCCCCCCCGCCACATTCCGAGAC-3′and 5′-GTCTCGGAATGTGGCGGGGGGGGCTGCGTAAATGAGAGTG-3′). Strains carrying these plasmids were grown in Luria Broth (LB) medium with 50 μg/ml kanamycin.

**Figure 1.**
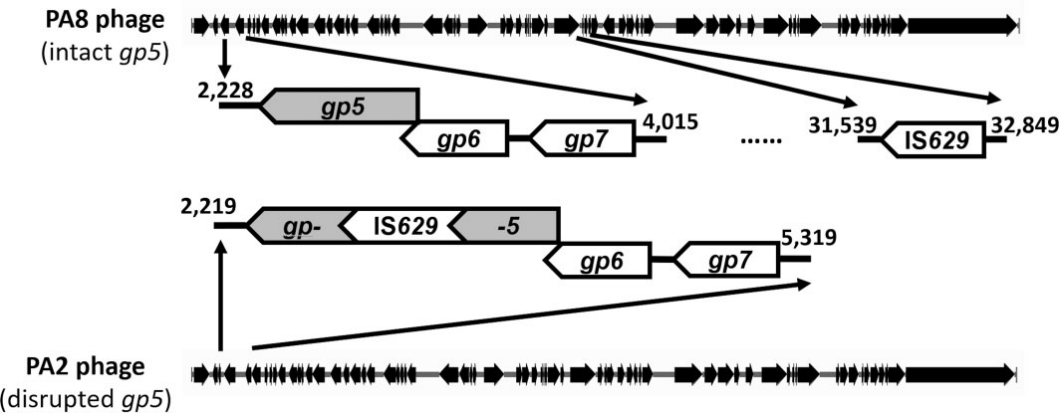
Genomic structures of PA8 phage and PA2 phage. Shown is a schematic representation of the genomes of ϕPA8 (top) and ϕPA2 (bottom). ϕPA8 has an intact *gp*5 gene that is proposed to encode an MT-A70 family methyltransferase and the IS*629* mobile element inserted into a non-coding region. In ϕPA2, the putative methyltransferase gene disrupted by the insertion of an IS*629* element. Also shown is the position of two putative coding sequences for PNB-1 (*gp*7) and PNB-2 (*gp*6) (see text) located upstream of the gene encoding the methyltransferase in both phages.

Examination of the ϕPA8 sequence in *Escherichia coli* PA8 revealed two ORF-encoding genes, directly upstream of the gene for M.EcoPA8orf6770P that encode proteins EcoPA8orf6775P and EcoPA8orf6780P, respectively. These ORFs are encoded by the 6^th^ (EcoPA8orf6775P) and 7^th^ genes (EcoPA8orf6780P) from the left end of the phage genome. Again, for simplicity, we will refer to these genes encoding EcoPA8orf6775P as *gp*6 and that EcoPA8orf6780P as *gp*7. To examine the potential role of these ORFs, we created two additional plasmids that were intended to enable overexpression and purification of the M.EcoPA8orf6770P alone or together with either one EcoPA8orf6775P or both EcoPA8orf6775P and EcoPA8orf6780P (see Figure 1). In all plasmids, the first gene was fused to a sequence encoding a His-tag to be used for nickel agarose column purification of the protein/protein complexes. In all cases, required DNA fragments were amplified by PCR from ϕPA8 genomic DNA with high-fidelity Platinum *PFX* DNA polymerase (Invitrogen, Thermo-Fisher, Waltham, MA). To construct the plasmid expressing the His-tagged methyltransferase protein (M.EcoPA8orf6770P), we used the following primers:

Forward primer 5′- GAACCCATGGCTAGCAGCCATC-3′,

Reverse primer 5′- CGACGGCCAGTGAATTGAC-3′.

To create the plasmid p-M.EcoPA8F1 that encodes the methyltransferase protein together with both EcoPA8orf6775P and EcoPA8orf6780P, we used the following primers: Forward primer 5′- GGGAATTCCATATGATGACAACTCAAGTTTCTG-3′, Reverse primer 5′- CGCGGATCCTCACTCTGACAATTCGTTGATC-3′. To create the plasmid p-M.EcoPA8F2 that encodes the methyltransferase protein together with EcoPA8orf6775P we used the following primers: Forward primer 5′- GGGAATTCCATATGTTGAAACAGATCGCTTTC-3′, Reverse primer 5′- CGCGGATCCTTATGCGATTGGTGAGGTG-3′. In all cases, the PCR reaction introduces restriction-sites for *NdeI* and *BamHI* (New England Biolabs, Beverly, MA) to the ends of the DNA fragments. For all plasmids, the PCR products were digested with these enzymes and ligated into pET-15b (MilliporeSigma, Burlington, MA) cut with the same enzymes.

The plasmid carrying only *gp*6 (EcoPA8orf6775P) (p-M.EcoPA8F2ΔM.EcoPA8) was created by first cutting the PCR amplified DNA containing *gp*5 and *gp*6 with *NdeI-HF* and *EcoRV* (New England Biolabs, Beverly, MA) to remove the *gp*5 (M.EcoPA8orf6770P) gene. pET-15b was digested with *Bam*HI and treated with Klenow fragment of DNA polymerase I (New England Biolabs, Beverly, MA) to create a 3′ blunt end for the ligation of *Eco*RV cut site on the digested insert. The vector was further digested by *Nde*I and ligated with the digested PCR product to create p-M.EcoPA8F2ΔM.EcoPA8 plasmid. The plasmids over-expressing His-tagged gene constructs were propagated in *E*. *coli* strain K802 (American Type Culture Collection, Manassas, VA, USA), purified, sequenced and subsequently transformed into *E*. *coli* strain BL21(DE3).

### *A. castellanii* killing assay

Measurements of the effect of STEC on *A. castellanii* viability was performed essentially as described (59). Briefly, bacterial prey strains were grown to saturation overnight in LB with 10mM MgSO_4_ at 30°C. For the strains carrying p-M.EcoPA8 or mutated p-M.EcoPA8 plasmid, 50 μg/ml kanamycin was added into the medium. The bacteria were harvested by centrifugation at 3000 × *g*, washed three times with equal volume fresh LB with 10mM MgSO_4_ and then suspended in equal volume of LB with 10mM MgSO_4_. The *A. castellanii* was prepared as described (59). 200 μl of prepared *A*. *castellanii* culture were harvested by centrifugation at 500 × *g* and re-suspended in 200 μl of washed bacterial culture, then co-cultured for 2 h at 30°C. After incubation, the *A*. *castellanii* viability was examined by trypan blue dye staining. The *A*. *castellanii* killing ability was calculated from number of dead cells divided by total cells, and the killing ability of lysogens were normalized to that of host strain MG1655 (background death rate). Each data point was determined from at least 15 biological replicates and each biological replicate was measured from at least 3 technical replicates.

### Measurement of spontaneous induction

Overnight cultures of *E. coli* lysogenized with either ϕPA2 or ϕPA8 were prepared and washed as described above. Control experiments revealed that washing reduced the amount of free phage to undetectable levels. The washed cells were diluted 1:40 into fresh LB containing 10 mM MgSO_4_ and incubated at 37°C. 50 μg/ml kanamycin was added into the medium for the strains carrying p-M.EcoPA8 plasmid. This time point was designated as time 0. At *t* = 0 and every 30 min subsequently 50 μl of culture samples were taken. Total DNA was extracted from each sample by adding an equal volume of Instagene matrix (BioRad, Carlsbad, CA). The isolated DNA was used as template for qPCR to quantify the amount of phage and bacterial DNA in each sample at various time points. The primers to quantify phage DNA were directed against *stx2A*: Forward primer 5′-ATTAACCACACCCCACCG-3′, Reverse primer 5′-GTCATGGAAACCGTTGTCAC-3′, TaqMan probe 5′-CAGTTATTTTGCTGTGGATATACGA-3′ labeled with fluorescent reporter dye HEX (hexacholoro-6-carboxyfluorescein) at the 5′ end and with the Black Hole Quencher 1 (BHQ 1) at the 3′ end. (Integrated DNA Technologies, USA). The primers used to quantify bacterial DNA were directed against the *uidA* gene:Forward primer 5′-GTGTGATATCTACCCGCTTCGC-3′, Reverse primer 5′- AGAACGGTTTGTGGTTAATCAGGA-3′, TaqMan probe 5′- TCGGCATCC GGTCAGTGGCAG T-3′ which was labeled with the fluorescent reporter dye FAM (fluorescein) at the 5′ end and with BHQ 1 at the 3′ end. The qPCR reactions were performed with Taq DNA polymerase (New England Biolabs, Beverly, MA) according to the manufacturer's instructions. 0.375 μM each of the forward and reverse primer plus 0.3 μM of the probe was used in the 20 μl qPCR reaction. The qPCR was conducted on a Bio-Rad MyIQ2 platform using the following program: 5 min at 95°C, 45 repeats of 10 s at 95°C, 45 s at 60°C.

Standard curves for qPCR were generated using DNA purified by phenol-chloroform extraction from isolated PA8 phage and MG1655. The MG1655::ϕPA8 lysogen was used as the source of phage DNA which was isolated by a modified PEG precipitation from ciprofloxacin-induced cultures (60). qPCR results were expressed as the ratio of total phage DNA:total bacterial DNA. Since the genomic DNA of each MG1655::ϕPAx lysogen contains only one prophage, ratios >1 denote the amount of phage released per bacterium. Each data point was determined from at least three biological replicates and each biological replicate was measured from at least three technical replicates.

### Reverse transcription

Overnight cultures of MG1655::ϕPA8 cells were prepared as described above, then diluted 1:40 into fresh LB with 10mM MgSO_4_ and incubated at 37°C for 2h. Cultures were harvested and total RNA was extracted using the hot acid phenol method with two modifications: (i) the 65°C incubation step was carried out for 10 min instead of 4 min; (ii) the extracted RNA was extracted with chloroform before ethanol precipitation. Residual DNA was removed by treatment with RNase-free RQ1 DNaseI (Promega, Madison, WI) according to the manufacturer's instructions. Primers (Forward primer 5′-CCGCTCGAGATGACAACTCAAGTTTCTG-3′, Reverse primer 5′-CAACTTCTTGGCGATGTTCC-3′) were used to create the cDNA from total mRNA of MG1655::ϕPA8. cDNA was prepared with RevertAid reverse transcriptase (Thermo-Fisher, Waltham, MA) following the manufacturers procedure. A no reverse transcriptase control was set up to rule out DNA contamination. The cDNA was examined by regular PCR with the same primer set (*T*_m_ = 59°C) and products were displayed on agarose gel. PA8 genomic DNA served as the positive control.

### RT-qPCR

Total RNA was extracted from the mid-log cultures grown as described above using Direct-zol RNA miniprep kit (Zymo Research, Orange, CA) with modifications: 1) After adding the Tri-reagent solution (Thermo-Fisher, Waltham, MA), samples were incubated for 15 min at room temperature; 2) Instead of on-column digestion, residual DNA was removed by RNase-free RQ1 DNaseI (Promega, Madison, WI) following the manufacturer's instructions. A cDNA library was created from total RNA with RevertAid reverse transcriptase (Thermo-Fisher, Waltham, MA) using random hexamer primers (Thermo-Fisher, Waltham, MA) following the manufacturer's instructions. The qPCR set-up was performed as described (61). Primer sets *uidA*F and *uidA*R and *stx2A*F and *stx2A*R were used to quantify *uidA* and *stx2A* mRNA, respectively using PowerUp SYBR Green Master Mix (Thermo-Fisher, Waltham, MA). qPCR was performed as described above. Statistical analysis of the data was performed as described below.

### Purification of phage-encoded methyltransferase protein (M.EcoPA8orf6770P) and protein complexes

Saturated overnight cultures of BL21(DE3) strains bearing the protein-producing plasmids were diluted 40-fold into LB supplemented with 100 μg/ml of ampicillin. At either OD_600_ 0.5 (cells bearing p-M.EcoPA8F1) or 0.8 (cells bearing p-M.EcoPA8F1, p-M.EcoPA8 or p-M.EcoPA8 ΔM.EcoPA8) 0.5 mM isopropyl-β-d-thiogalactopyranoside (IPTG) was added to the cultures to induce protein expression. The induced cultures were further incubated at 18°C for 12h and then harvested by centrifugation at 3000 × *g* for 30 min. All subsequent procedures were performed at 4°C or on ice. The cell pellet from 1L of induced culture was re-suspended in 10ml lysis buffer (20mM sodium phosphate pH 7.4, 300 mM NaCl, 0.1% Tween20 v/v). A protease inhibitor cocktail (5 μg/ml of leupeptin, 50 μg/ml benzamidine, 10 U of aprotinin, 5 μg/ml of pepstatin, and 5 μg/ml tosyl sulfonyl phenylalanyl chloromethyl ketone) were added and the resuspended cells were lysed by passing twice through a French press at 20 000 psi. The lysate was diluted 5-fold with lysis buffer. The diluted lysate was centrifuged at 20 000 × *g* for 40 min and then re-spun at 20 000 × g for 20 min to remove cell debris. The supernatant was loaded on 20 ml HisPur Ni-NTA resin (Thermo-Fisher, Waltham, MA) equilibrated with lysis buffer. The column was washed sequentially with 100 ml lysis buffer, 100 ml lysis buffer with 50 mM imidazole, 60 ml lysis buffer with 100 mM imidazole and 30 ml lysis buffer with 150 mM imidazole. The proteins were eluted with lysis buffer plus 250 mM imidazole. A portion of the eluate was concentrated using a 10K MWCO centrifugal device (Macrosep advance, Pall Corporation) and immediately assayed for methyltransferase activity. The balance of the eluate was dialyzed against lysis buffer without imidazole and stored at 4ºC or dialyzed against stock buffer (20 mM sodium phosphate pH 7.4, 100 mM NaCl, 20% of glycerol v/v) and flash frozen and stored at −80ºC. All the purified proteins were checked on SDS-PAGE gels to determine the level of purity. Protein concentrations were measured by Bio-Rad protein assay (Bio-Rad, Carlsbad, CA) following the company's procedure to determine the yield of the proteins.

### Methyltransferase activity assay

Methyltransferase activity was monitored using the SAM510: SAM Methyltransferase Assay kit from G-Biosciences (St. Louis, MO USA). Assays were performed following the manufacturer's instructions with modifications to minimize the amounts of enzyme needed in the assay. DNA, RNA or ribosomal subunits were resuspended in SAM assay buffer without MnCl_2_ (0.1 M pH 8.0 Tris). For each assay, either 27 μg of total DNA, 27 μg of total RNA, 27 μg of high-molecular weight RNA, 27 μg of low molecular weight RNA, 0.42 μM of 16s or 23s rRNA or 0.42 μM of 30S or 50S ribosomal subunits were added to individual wells of a 96 well plate. Subsequently 20 μl of protein buffer (20 mM sodium phosphate pH 7.4, 300 mM NaCl, 0.1% Tween20 v/v, 250 mM imidazole) without or with 4.8 μg methyltransferase protein or 7.2 μg the purified methyltransferase-PNB-2 complex was added to each well. Where added, the final protein concentration was 1.75 μM. Next SAM assay buffer without MnCl_2_ was added to bring to reaction volume into 115 μl. The plate was shaken for 10s on the plate reader for mixing and warmed to 37°C. Subsequently 5.5 μl of freshly prepared chilled SAM Mix (each 110.5 μl SAM mix contains: 50 μl SAM Enzyme Mix, 30 μl SAM Colorimetric Mix, 30 μl 6.9 mM S‐Adenosylmethionine, 0.5 μl 1 M MnCl_2_ in 0.1 M pH 8.0 Tris) was added and mixed on the plate reader to start the reaction. During a 100 min incubation in 37°C, the OD at 510 nm was measured every 2 min by a plate reader (see Supplementary Figure S1). A blank containing only protein buffer was subtracted from experimental samples. Velocity of SAM consumption was calculated as described by the manufacturer and plotted as amount of methyl groups transferred per minute by 1 μmol of enzyme (M.EcoPA8orf6770P or the holoezyme). Background methyltransferase activity of the enzyme alone in the absence of any added methyl group acceptor was subtracted from the plus substrate values (see also Supplementary Figure S1). Measurements were made on at least 3 biological replicates and each biological replicate was measured from at least three technical replicates.

***Methyltransferase substrate preparation*** Total DNA was purified from bacterial cells by phenol/chloroform/isoamyl alcohol extraction method followed by a chloroform extraction and precipitation with 2 volume of isopropanol and 0.1 volume of 3 M sodium acetate. Total RNA was purified by the hot phenol method as described above. The size exclusion separation of 16S and 23S rRNAs from purified total RNA was performed using Sephacryl S-100 HR resins (GE Health, Piscataway, NJ) following the manufacturer's instructions. The fractions containing RNA were lyophilized. Ribosomes were prepared by ultra-centrifugation followed by a gradient ultracentrifugation. In brief, 600 ml of mid-log culture (OD_600_ = 0.6) was harvested by centrifugation at 3000 × *g* for 15 min, washed with 25 ml S100 buffer (30 mM Tris pH 7.9, 30 mM MgCl_2_, 140 mM KCl, 6 mM BME) in the presence of protease inhibitor cocktail and resuspended in 25 ml S100 buffer. All subsequent procedures were performed at 4°C or on ice.

The resuspended cells were lysed by passing through a French press twice as described above. The lysate was clarified by centrifugation at 15 000 × *g* for 15 min at 4°C twice. Ribosomes were collected by ultra-centrifugation at 32 700 × *g* for 4h at 4°C. The collected ribosomes were flash frozen and stored at –80ºC. For use, the ribosome pellet was suspended in 5 ml 30–50 Buffer A (50 mM Tris pH 7.0, 10.5 mM MgCl_2_, 100 mM NH_4_Cl, 6 mM BME) and subunits separated by velocity gradient sedimentation on 10–30% sucrose (v/v) gradients by centrifugation at 25 000 × g for 14 h at 4°C in a Beckman SW28 rotor. Gradient fractions were separated on Gradient Station spv1.5 (BioComp, Canada) while scanning at 254 nm and stored at –80ºC. Where desired, rRNA was extracted from the gradient fractions containing 30S or 50S ribosome subunits by a modified phenol/chloroform extraction. First, one volume of phenol pH 8.0 was added to the fractions then vortexed for 1 min prior to 5 min centrifugation at 14 000 × *g*. The extraction step was repeated with 1 volume of acid phenol/chloroform (5:1) pH 4.5 and with 1 volume of chloroform. The extracted rRNA was precipitated by isopropanol precipitation followed by one 70% isopropanol wash.

### Crosslinking of M.EcoPA8orf6770P-EcoPA8orf6775P complex

Cross-linking was performed as described by Finger and Richardson (62) with minor modifications. Briefly, dimethyl suberimidate (DMS) buffer (160 mM triethanolamine–HCl pH 8.0, 20 mM magnesium acetate, 2 mM EDTA, 0.2 mM dithiothreitol) and cross-linking buffer (60 mM triethanolamine–HCI pH 8.0, 7.5 mM magnesium acetate, 0.75 mM EDTA, 0.75 mM dithiothreitol) were freshly made on ice. 10 mg of DMS was then added to the 0.15 ml DMS buffer and mixed thoroughly. Experimental samples contained 0.12 mg of co-purified His-tagged M.EcoPA8orf6770P-EcoPA8orf6775P complex in 27 μl cross-linking buffer, reaching a concentration of 0.1nM. As controls, 0.48 mg (0.69 μM) His-tagged methyltransferase protein (M.EcoPA8orf6770P) or 1.92 mg (3.8 μM) His-tagged EcoPA8orf6775P were used. Once the protein was placed into the crosslinking buffer, the reaction was started by adding 3 μl freshly made DMS solution. Following a 15 min incubation at 23°C, the reactions were quenched by adding 3 μl of 1.4 M ethanolamine. Subsequently 3 μl of 10× Laemmli loading buffer (625 mM Tris–HCl pH 6.8, 20% sodium dodecyl sulfate (SDS, v/v), 0.5% bromophenol blue v/v, 10% glycerol v/v, 0.5% β-mercaptoethanol v/v) was added and the reactions products were resolved on an 13% SDS-PAGE gel which was prepared for immunoblotting. The proteins were transferred to a 0.45μm Hybond® P Western blotting PVDF membranes (Amersham Biosciences UK) at 4°C, and the membrane was washed with TBST (20 mM Tris pH 7.5, 0.001% Tween 20, 150mM NaCl) 3 times and blocked with filtered 3% BSA (v/v) in TBST on shaker at room temperature for 1 h. The membrane was exposed to primary antibody (6x-His tag antibody HIS.H8, Invitrogen, Thermo-Fisher, Waltham, MA) in TBST at a 1:2000 ratio on shaker at room temperature for 1 h. Following three washes with TBST, the membrane was exposed to the secondary rabbit anti-mouse antibody (Thermo-Fisher, Waltham, MA) on shaker at room temperature for 1 h. At last, after another three washes with ddH_2_O, the membrane was developed with Clarity Western ECL substrate (Bio-Rad) and was imaged with ChemiDoc XRS + imaging system (Bio-Rad).

### Statistical analysis

Error bars in the figures represent standard deviations of multiple biological replicate experiments. To determine the statistical significance between different samples, all data were analyzed using GraphPad PRISM 4.0 software by unpaired t-test or nonpaired, or one-way analysis of variance (one-way ANOVA) followed by Tukey's multiple comparisons test.

## Results

### Phage functions control STEC virulence

Despite the strong sequence similarity of their Stx-encoding prophages (>96.9% identity), STEC O157:H7::ϕPA2 or STEC O157:H7::ϕPA8 produce different levels of Stx (63), the essential virulence factor of STEC. The major sequence difference between ϕPA2 and ϕPA8 is the location of an IS*629* insertion element (Figure 1). In ϕPA8 the IS*629* is located in an intergenic region. In ϕPA2, the IS*629* element disrupts a gene (*gp*5, see Figure 1) predicted to encode a nucleic acid methyltransferase (see Supplementary Figure S2). These observations suggest that the putative methyltransferase may affect the relative virulence of these two STEC strains.

**Figure 2.**
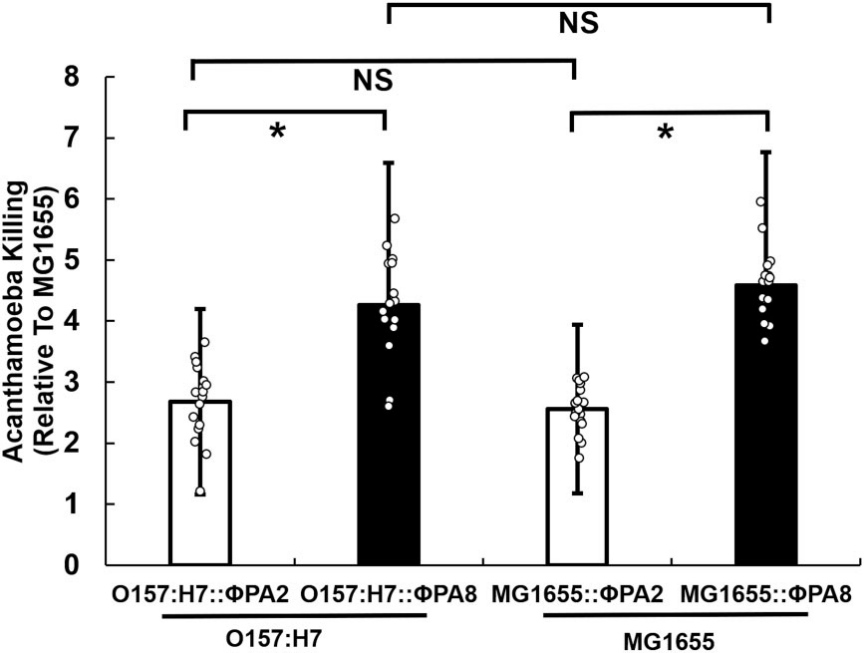
Amoeba killing efficiency of *E. coli* bearing ϕPA2 or ϕPA8, *Acanthamoeba castellanii* were separately co-cultured with *E. coli* strains bearing either ϕPA2 or ϕPA8. After 2h, the fraction amoebae killed by phage-bearing strains was measured relative the number of amoebae killed in co-cultures with naïve MG1655. The left side shows the results obtained with STEC O157:H7::ϕPA2 and STEC O157:H7::ϕPA8. The right side fraction of amoeba killed in co-coltures with MG1655::ϕPA2 and MG1655::ϕPA8. Error bars represent standard deviations determined from at least 15 biological replicates and each biological replicate was measured from at least 3 technical replicates. *P < 0.05; NS: not significant *P* > 0.05.

To begin to test this idea, we examined the virulence of the STEC O157:H7 strains bearing ϕPA2 or ϕPA8 by analyzing their ability to kill the model protist predator *Acanthamoeba castellanii* (64,65). Consistent with previous observations, we find that these two Stx-encoding strains both kill amoebae, however their killing efficiencies differ (Figure 2, left); STEC O157:H7::ϕPA8 kills amoebae more efficiently than does STEC O157:H7::ϕPA2.

Analysis of the genome sequences of STEC O157:H7::ϕPA2 and O157:H7::ϕPA8 suggests that these strains contain multiple additional prophages in addition to ϕPA2 and ϕPA8, respectively. Moreover, the host genomic sequences of these two strains, while highly similar, are not completely identical. Therefore, to determine if the sequence differences between ϕPA2 and ϕPA8 are solely responsible for the differences of these two STEC strains, we repeated the amoeba killing experiments with lysogens formed by ϕPA2 and ϕPA8 in the *E. coli* strain MG1655. The amoeba killing efficiencies of the MG1655 lysogen strains (Figure 2, right) mirror that of the two STEC O157:H7 strains. Thus, amoeba killing efficiency is independent of host strain background, meaning the differences in STEC-mediated cell killing depends solely on the differences between two the Stx-encoding prophages.

### A putative methyltransferase regulates STEC virulence

**Figure 3.**
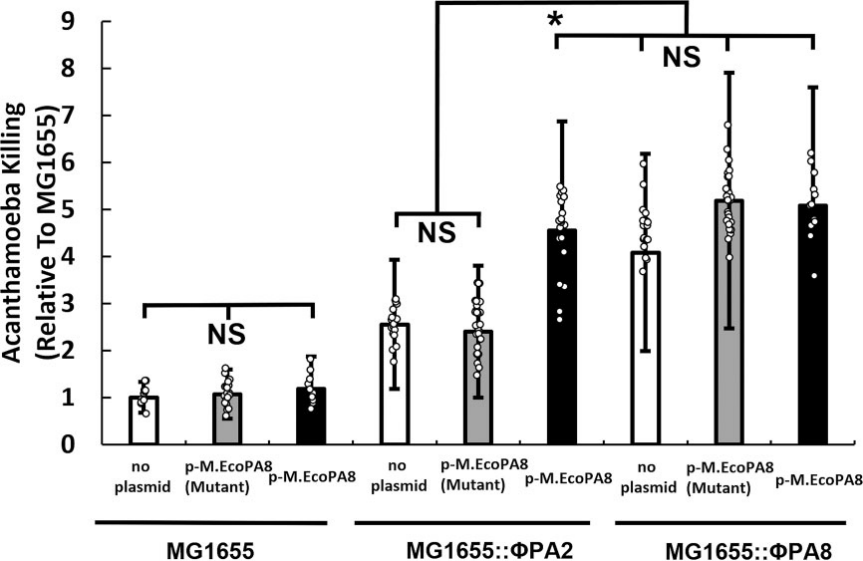
The conserved active site motif is essential to the effects of M.EcoPA8orf6770P on STEC virulence. *E. coli* strains bearing either no Stx-encoding prophage (MG1655, left panel), ϕPA2 (MG1655:: ϕPA2, middle panel) or ϕPA8 (MG1655:: ϕPA8, right panel) and bearing either a control plasmid (white bars), a plasmid encoding the DPPW→APPA mutant (gray bars) or wild-type M.EcoPA8orf6770P (black bars) were co-cultured with *A. castellanii*. Shown is the amoeba killing efficiency of these strains. Error bars represent standard deviations determined from at least 15 biological replicates and each biological replicate was measured from at least three technical replicates. *P < 0.05; NS: not significant *P* > 0.05.

The main difference between the sequences of ϕPA2 and ϕPA8 is the disruption of the *gp*5gene in ϕPA2 that is predicted to encode an adenine methyltransferase M.EcoPA8orf6770P. To determine whether the disruption causes the decreased virulence of MG1655::ϕPA2 and STEC O157:H7::ϕPA2, we attempted to complement the loss of the methyltransferase using a plasmid (p-M.EcoPA8) that constitutively expresses a low level of the putative methyltransferase (see Methods and Materials). We introduced this plasmid into MG1655::ϕPA2 and measured the efficiency with which the plasmid-bearing strain kills amoebae. We find that carriage of the methyltransferase-encoding plasmid increases the killing efficiency of MG1655::ϕPA2 to a level that is similar to that of MG1655::ϕPA8 (Figure 3, middle white & black bars vs right white bars, *P* < 0.05). Introducing the methyltransferase-encoding plasmid into either MG1655 or MG1655::ϕPA8 has no significant effect on the amoeba killing efficiency of these two strains (Figure 3, right, *P* > 0.05).

Most methyltransferases act by transferring a methyl group from *S*-adenosyl-l-methionine (SAM) to a substrate, modifying the biological activity of that substrate. Analysis of the putative methyltransferase protein sequence revealed the presence of a conserved DPPW motif in M.EcoPA8orf6770P (66) (see Supplementary Figure S2). This motif comprises the active site of MT-A70 methyltransferases site (67,68). To test whether the predicted methyltransferase activity plays a role in mediating virulence, we mutated the sequence of this motif from DPPW→APPA. These changes are predicted to block the methyltransferase activity (67,68)

Unlike the plasmid bearing the wild-type version of the putative methyltransferase gene, the plasmid bearing inactivated methyltransferase gene fails to complement the decreased virulence of MG1655::ϕPA2 (Figure 3, grey bars). This finding indicates that the putative methyltransferase enables the increased virulence of bacterial strains bearing Stx-encoding prophage ϕPA8. It is possible the DPPW→APPA mutation exerts its effect by destabilizing the protein. Although we have not definitively tested this alternative, the DPPW→APPA mutant protein accumulates to similar levels as wild-type and identical mutations of in other MT-A70 methyltransferases did not affect protein stability (e.g. 69,70).

### Phage encoded methyltransferase regulates prophage spontaneous induction frequency

**Figure 4.**
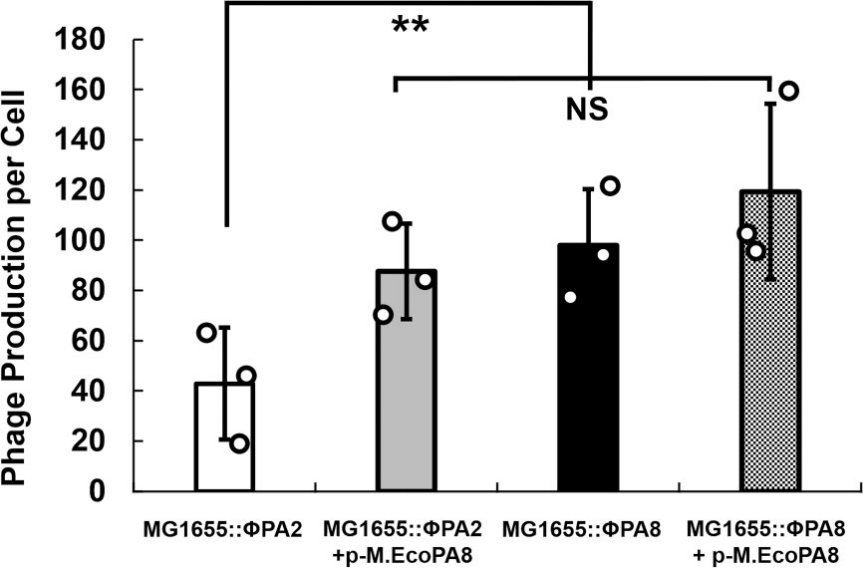
M.EcoPA8orf6770P increases the frequency of prophage spontaneous induction. Overnight cultures of *E. coli* strains MG1655::ϕPA2 and MG1655::ϕPA8 either do or do not contain p-M.EcoPA8, the plasmid encoding the putative phage methyltransferase, were diluted 50-fold and grown for 2h. Subsequently the number of *E coli*. cells and free phages were measured by qPCR as described in Methods and Materials. Shown is the number of phages produced per *E. coli*. Error bars represent standard deviations determined from 3 biological replicates and each biological replicate was measured from at least three technical replicates. **P < 0.01; NS: not significant *P* > 0.05.

Bacterial strains bearing Stx-encoding prophages produce and release significant amounts of Stx2 only during lytic growth of the phage (31,71,72). Because *E. coli* strains containing either ϕPA2 and ϕPA8 can kill amoeba in the absence of added inducer (Figure 2), we hypothesize that the varying levels of virulence between these strains are likely due to differences in the frequency of spontaneous induction of these prophages. Since the virulence difference can be eliminated by ectopically expressing the M.EcoPA8orf6770P methyltransferase in ϕPA2-containing strains (Figure 3), we also suggest this gene product is regulating prophage induction. To test these ideas, we monitored the amount of phage spontaneously released from MG1655::ϕPA2 and MG1655::ϕPA8 after two hours of growth, i.e. the length of time these strains are incubated with predator amoeba (Methods and Materials). We found that MG1655::ϕPA8 spontaneously releases significantly larger amounts of phages as compared to MG1655::ϕPA2 (Figure 4, white bar vs black bar, *P* < 0.01), indicating that the higher amoeba killing efficiency of MG1655::ϕPA8 is a result of increased Stx production and release by this strain. Consistent with this suggestion, RT-qPCR measurements indicate MG1655::ϕPA2 contains ∼5-fold less *stx*2A mRNA than does MG1655::ϕPA2 (Supplementary Figure S3). We have not yet measured *stx*2A mRNA levels in STEC O157:H7 strains bearing ϕPA2 or ϕPA8, but based on the observations of others (63 and Ed Dudley, personal communication) we anticipate similar results will be obtained with those strains.

The methyltransferase-encoding plasmid p-M.EcoPA8 into MG1655::ϕPA2 complements the disruption of *gp*5 (Figure 4, gray bar), increasing the number of phage released by the strain to an amount similar to that produced by MG1655::ϕPA8. Introducing the same plasmid into MG1655::ϕPA8 has no significant effects on induction frequency (*P* > 0.05). The prophage induction frequencies (Figure 4) of MG1655::ϕPA8, MG1655::ϕPA2 and MG1655::ϕPA2 + p-M.EcoPA8 mirrors the virulence of these strains (Figure 3), again indicating that the putative methyltransferase affects prophage virulence by regulating induction frequency and modulating the amount of cytolethal Stx produced by the bacteria.

### The purified putative M.EcoPA8orf6770P alone does not display significant specific methyltransferase activity

**Figure 5.**
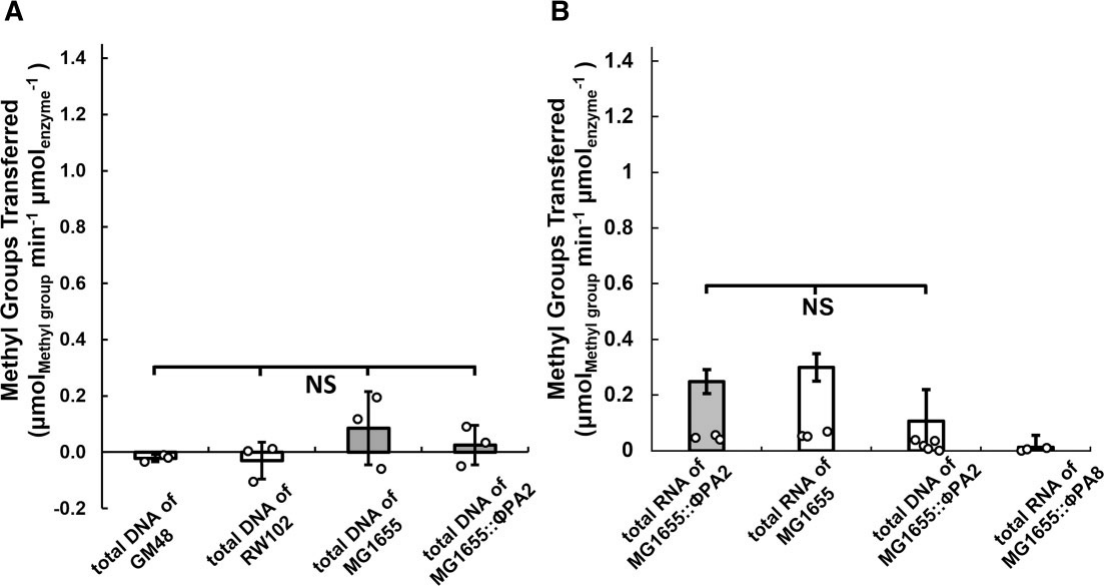
M.EcoPA8orf6770P alone does not display significant methyltransferase activity. (**A**) Purified M.EcoPA8orf6770P was separately incubated with total DNA isolated from *E. coli* strains GM48 (*dam*^−^, *dcm*^−^), RW102 (*dam*^−^), MG1655 (*dam*^+^, *dcm*^+^) or MG1655::ϕPA2 (*dam*^+^, *dcm*^+^*gp*5^−^) as indicated. (**B**) The M.EcoPA8orf6770P was separately incubated with total RNA or DNA isolated from MG1655, MG1655::ϕPA2 or MG1655::ϕPA8 as indicated. The consumption of SAM was measured as described in Materials and methods and plotted as the amount of methyl groups transferred per min per μmol of enzyme used. Error bars represent standard deviations that are determined from at least three biological replicates and each biological replicate was measured from at least three technical replicates. **P < 0.01; NS: not significant *P* > 0.05.

The M.EcoPA8orf6770P protein in ϕPA8 is annotated as an adenine DNA methyltransferase (56), and our findings in Figure 3 are consistent with it having methyltransferase activity. Moreover, many methyltransferases similar to M.EcoPA8orf6770P are annotated in REBASE as GATC N6 adenine DNA methyltransferases (73). In addition, a similar protein encoded by a related Stx-encoding phage found in a STEC O104 strain can methylate GATC sequences in DNA *in vivo* when overproduced from a plasmid (74). To confirm M.EcoPA8orf6770P has methyltransferase activity and to begin to identify its substrate, we overexpressed and purified this putative methyltransferase (details in Materials and Methods) and used a colorimetric assay (Methods and Materials) to measure its methyltransferase activity using S-adenosyl-L-methionine and potential substrates. We found that in the presence of DNA isolated from *dam*^−^ and *dam*^−^/*dcm*^−^ strains, SAM utilization activity of M.EcoPA8orf6770P is nearly indistinguishable from background (Figure 5A, also Supplementary Figure S1). We also examined SAM utilization activity of M.EcoPA8orf6770P in the presence of DNA isolated from MG1655 or MG1655::ϕPA2 strains. These strains lack the gene encoding the putative methyltransferase, and thus nucleic acids isolated from them would not bear a methyl group at the potential target site of M.EcoPA8orf6770P. However, the SAM utilization activity of M.EcoPA8orf6770P on these substrates is also nearly indistinguishable from background (Figure 5A, also Supplementary Figure S1). These findings indicate that M.EcoPA8orf6770P does not display GATC-specific DNA methyltransferase activity *in vitro*, nor does it display significant methyltransferase activity with any other DNA substrate under our conditions *in vitro*. We also examined the ability of M.EcoPA8orf6770P to use RNA isolated from MG1655, MG1655::ϕPA2 or MG1655::ϕPA8 as a substrate and compared the SAM utilization activity seen when DNA from MG1655::ϕPA8 is used as a substrate. Although the average SAM utilization activity in the presence of RNA is slightly higher, there is no statistically significant difference in activity of M. EcoPA8orf6770P when presented with either RNA and DNA or between RNA isolated from various sources (Figure 5B) M.EcoPA8orf6770P showed significantly lower methyltransferase activity in the presence of RNA from MG1655::ϕPA8. However, the SAM utilization activity of M.EcoPA8orf6770P in the presence of RNA from MG1655::ϕPA8 was identical to background. These results show that *in vitro* M.EcoPA8orf6770P displays weak and non-substrate specific SAM utilization activity in the presence of DNA or RNA.

### The putative M.EcoPA8orf6770P methyltransferase forms a complex with another protein

Full and specific activity of enzymes in the MT-A70 superfamily typically require formation of a heteromer(s) with another subunit(s) (75). Thus, the weak, nonspecific SAM utilization activity of the M.EcoPA8orf6770P subunit seen in the presence of RNA or DNA *in vitro* suggests that another subunit may be needed to confer substrate specificity. Analysis of the DNA sequence adjacent to gene encoding the putative methyltransferase identified two upstream genes *gp*6 and *gp*7. These two ORFs encode the hypothetical proteins EcoPA8orf6775P and EcoPA8orf6780P, respectively. RT-PCR analysis detects a transcript on which the genes encoding these ORFs are co-transcribed with *gp*5 (Supplementary Figure S4, lane 2 and lane 3). Additionally, preliminary results from RNAseq experiments indicate that the genes *gp*6 and *gp*5 that encoding EcoPA8orf6775P and the putative methyltransferase, respectively, are co-transcribed from a promoter that is induced at approach to stationary phase (Berger, P., personal communication). This analysis also indicates that *gp*7, the gene encoding EcoPA8orf6780P, is not co-induced under this condition. Regardless and intriguingly, structure-based functional predictions of the two upstream ORFs suggests that each ORF contains a different class of winged HTH nucleic acid binding motif. To reflect this finding, we will henceforth refer to EcoPA8orf6780P, the product of the *gp*7 gene as PNB-1 (putative nucleic acid binding protein-1) and EcoPA8orf6775P, the product of the *gp*6 gene as PNB-2 (putative nucleic acid binding protein-2).

**Figure 6.**
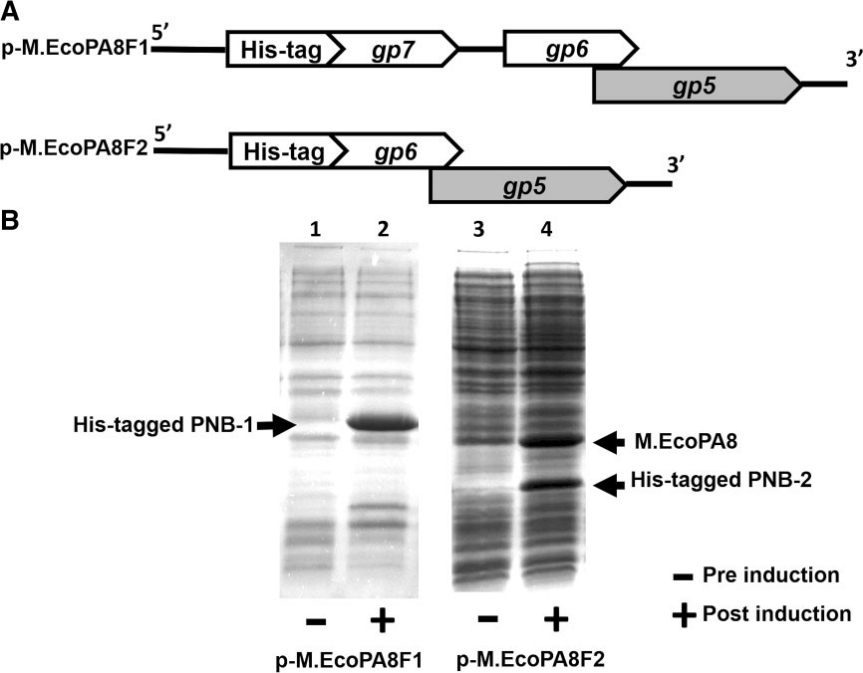
The M.EcoPA8orf6770P and PNB-2 proteins are co-expressed. (**A**) Shown are schematics of the coding regions of two plasmids p-M.EcoPA8F1 and p-M.EcoPA8F2, designed to co-express either His-tagged PNB-1 along with PNB-2 and the putative methyltransferase (M.EcoPA8orf6770P) (top) or His-tagged PNB-2 and the putative methyltransferase (M.EcoPA8orf6770P) (bottom), respectively. (**B**) Cells bearing these plasmids were induced by IPTG and lysed as described in the method. Shown is a photograph of a Coomassie Blue stained SDS-PAGE gel of the whole cell lysates of the *E. coli* cells bearing p-M.EcoPA8F1 or p-M.EcoPA8F2 that either were (+) or were not (–) exposed to 0.5 mM IPTG during the mid-log phase of growth. The labels indicate the positions of the M.EcoPA8orf6770P (M.EcoPA8), His-tagged PNB-1 proteins and His-tagged PNB-2 proteins.

To determine whether the phage-encoded putative M.EcoPA8orf6770P methyltransferase requires an additional subunit for activity, we attempted to determine whether the product of *gp*6, without or with that of *gp*7 (i.e. PNB-2 and PNB-1, respectively) form a complex with the methyltransferase. For this experiment, we two constructed plasmids p-M.EcoPA8F1 and p-M.EcoPA8F2. The plasmid p-M.EcoPA8F1 utilize the T7 RNA polymerase promoter to direct transcription of *gp*6 and *gp*7 together with the putative methyltransferase gene *gp*5, whereas in p-M.EcoPA8F2 the T7 RNA polymerase promoter controls transcription of *gp*6 together with the putative methyltransferase gene *gp*5. In each plasmid, a DNA sequence encoding an N-terminal His-tag is fused to the upstream-most ORF in the construct, i.e. PNB-1 (EcoPA8orf6780P, *gp*7) or PNB-2 (EcoPA8orf6775P, *gp*6), in p-M.EcoPA8F1 and p-M.EcoPA8F2, respectively. (Figure 6A, see Methods and Materials for details). This placement allows us to co-purify any proteins associated with the His-tagged protein.

Cells bearing p-M.EcoPA8F1 overproduced PNB-1 (EcoPA8orf6780P) in the presence of IPTG. Surprisingly we did not observe co-expression of either of the two genes downstream from PNB-1 (Figure 6B). This observation may be explained by the predicted presence of a *Rho*-independent transcription terminator immediately downstream of *gp*7:

*gp* 7→ ^3335^ TCCCCTACCAT CCCCGAC ATCCC GTCGGGG TTTTCATATCTG ^3294^ → *gp* 6

**Figure 7.**
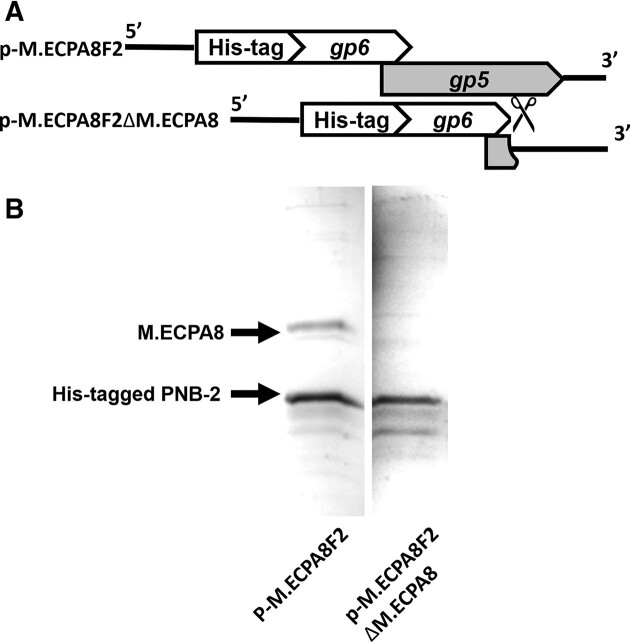
Co-purification of PNB-2 and M.EcoPA8orf6770P proteins. Proteins were isolated from lysates of *E. coli* bearing either the p-M.EcoPA8F2 or p-M.EcoPA8F2ΔM.EcoPA8 plasmids (**A**) using NTA-agarose (see Methods and Materials) and displayed on SDS-PAGE gel. (**B**) Proteins were visualized by Coomassie Blue staining. The expected positions of the M.EcoPA8orf6770P (M.EcoPA8) and His-tagged PNB-2 proteins are indicated.

In contrast, the putative methyltransferase and PNB-2, the product of *gp*6, are co-expressed when IPTG was added to mid-log cultures of BL21(DE3) cells bearing p-M.EcoPA8F2 (Figure 6B, right lane). When cell extracts of this strain are bound to a nickel-NTA agarose column, we found that a protein identical in size to the putative methyltransferase co-purified with the His-tagged PNB-2 protein (Figure 7B left lane). This finding suggests that putative methyltransferase associates with PNB-2. To confirm this finding, we modified p-M.EcoPA8F2 so that it no longer contains the coding sequence for the putative methyltransferase and repeated the purification (Figure 7A, p-M.EcoPA8F2ΔM.EcoPA8 plasmid). We found that without the cloned putative methyltransferase gene on the plasmid, no other proteins co-purify with PNB-2 (Figure 7B right lane). This finding confirms that the complex was formed by the putative M.EcoPA8orf6770P methyltransferase and PNB-2.

To extend these findings, we used cross-linking to examine the protein content of the putative methyltransferase-PNB-2 complex. We found that adding cross-linker to the putative methyltransferase-PNB-2 complex results in the formation of a species that has a molecular weight consistent with the formation of a 1:1 complex between one putative methyltransferase subunit and one PNB-2 subunit (Supplementary Figure S5), suggesting that the methyltransferase holoenzyme consists of a one-to-one ratio of these two subunits. We found that the purified holoenzyme was very unstable, the PNB-2 subunit in particular. Therefore we are unable to confirm the existence of this complex by other methods that require longer incubation times.

### The methyltransferase-PNB-2 holoenzyme uses RNA as a substrate

**Figure 8.**
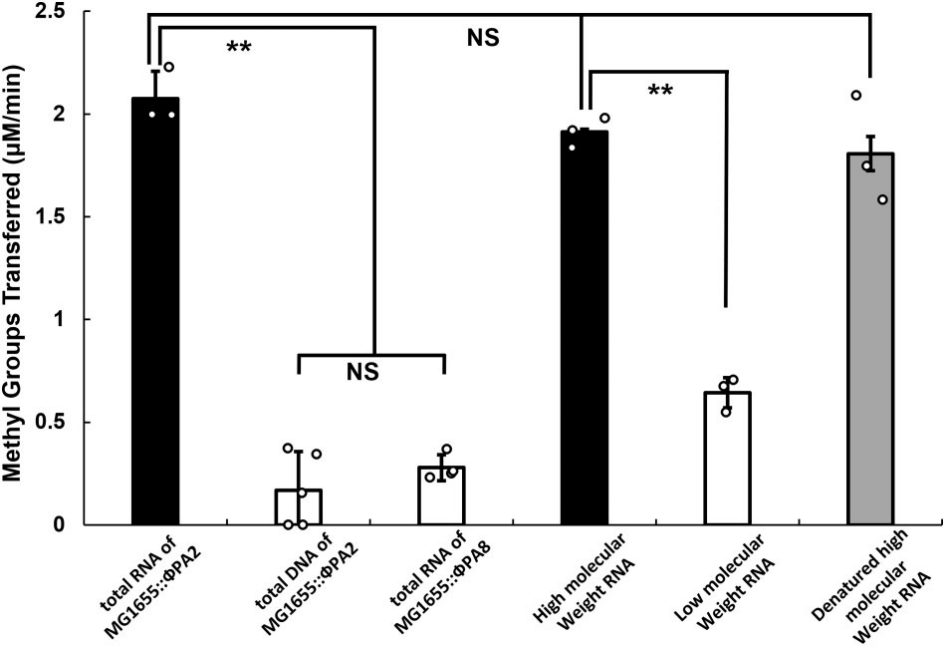
M.EcoPA8orf6770P-PNB-2 holoenzyme is a ribosomal RNA methyltransferase. Purified M.EcoPA8orf6770P-PNB-2 complex was incubated with the indicated potential substrates and the consumption of SAM was measured as described in Methods and Materials and plotted as the amount of methyl group transferred per min per μmol of enzyme used. Error bars represent standard deviations that are determined from at least 3 biological replicates and each biological replicate was measured from at least 3 technical replicates. **P < 0.01; NS: not significant *P* > 0.05.

In order to examine whether the putative M.EcoPA8orf6770P-PNB-2 complex comprises a specific methyltransferase holoenzyme and, if so, identify the substrate(s) that is methylated by the complex, we initially compared the methyltransferase activity of the purified M.EcoPA8orf6770P-PNB-2 complex in the presence of either total DNA or total RNA isolated from MG1655::ϕPA2. We chose to use nucleic acids isolated from this strain since it lacks the gene encoding the putative methyltransferase, and thus substrates isolated from it would not bear a methyl group at the target site of the putative holoenzyme. Our results show that the SAM utilization by the M.EcoPA8orf6770P-PNB-2 complex was > 13.5-fold higher when MG1655::ϕPA2 total RNA, rather than MG1655::ϕPA2 total DNA, was added as a substrate (Figure 8, *P*< 0.01). Moreover, when the RNA isolated from MG1655::ϕPA8 was used, the SAM utilization activity of the complex is not significantly different than when DNA from MG1655::ϕPA2 was added (Figure 8, *P*> 0.05). These results indicate that the complex preferentially uses RNA, not DNA as a substrate. Additionally, comparing the results in Figures 5 and 8 shows that PNB-2 confers substrate specificity to the holoenzyme, and that this subunit directs M.EcoPA8orf6770P to specifically use RNA as a substrate. Consistent with this idea, both Alphafold (76,77) and threading analyses (78) suggest that the structure of the PNB-2 subunit contains a nucleic acid binding motif.

We wished to identify the type of RNA preferred by the holoenzyme. Considering that the rRNAs and tRNAs make up 95–97% of the total RNA isolated from bacterial cells (79), we first tested whether RNAs in these classes could serve as a substrate for the holoenzyme. Therefore, as a first step, we size fractionated total RNA isolated from host MG1655. Although the high and low MW fractions may contain trace amounts of mRNA (80), these classes should predominantly consist of 16S and 23S rRNAs or tRNAs, respectively (81). We examined the ability of various size classes of RNA to serve as a substrate for the holoenzyme. We found that the holoenzyme showed the highest SAM utilization activity when incubated with high molecular weight fractions of RNA isolated from the host MG1655 strain (Figure 8, right black bar). Using high MW RNA fraction that had been boiled and rapidly cooled, a treatment that should disrupt residual rRNA secondary structure, as a substrate did not affect methyltransferase activity (Figure 8, right grey bar) suggesting that M.EcoPA8orf6770P-PNB-2 primarily recognizes RNA sequence(s).

### M.EcoPA8orf6770P-PNB-2 holoenzyme prefers purified and ribosome-associated 16S ribosomal RNAs as substrates

**Figure 9.**
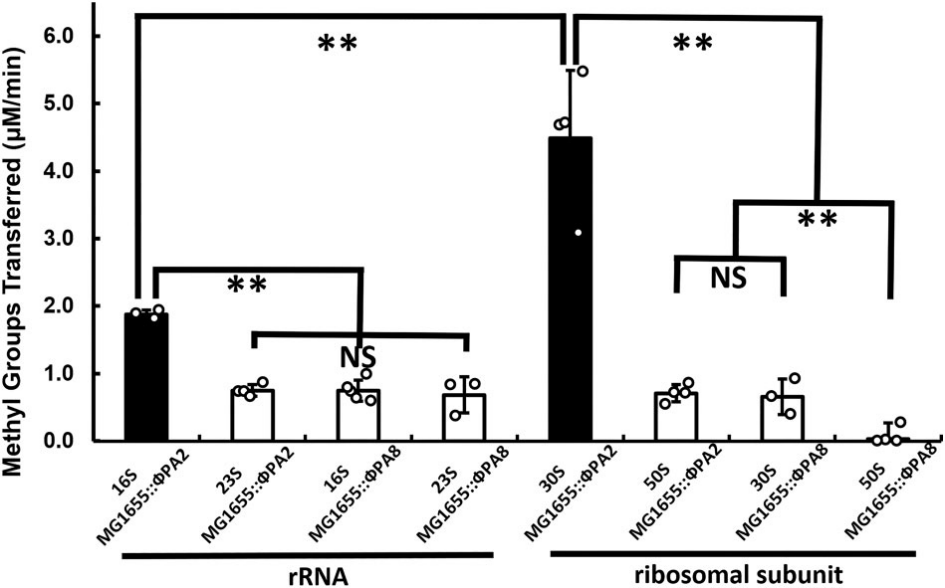
The M.EcoPA8orf6770P-PNB-2 holoenzyme preferentially methylates the 16S rRNA. 16S rRNA and 23S rRNA (left panel) or 30S and 50S ribosomal subunits (right panel) were purified from MG1655::ϕPA2 and MG1655::ϕPA8 strains and separately incubated with M.EcoPA8orf6770P-PNB-2 holoenzyme. The consumption of SAM was measured as described in Methods and Materials and plotted as the amount of methyl group transferred per min per μmol of enzyme used. Error bars represent standard deviations that are determined from at least three biological replicates and each biological replicate was measured from at least 3 technical replicates. **P < 0.01; NS: not significant *P* > 0.05.

Since ribosomal RNA comprises > 90% of the RNA molecules in the high molecular weight RNA fraction, we hypothesized that the M.EcoPA8orf6770P-PNB-2 methyltransferase holoenzyme is targeting rRNA. We tested this by measuring the methyltransferase activity of M.EcoPA8orf6770P-PNB-2 holoenzyme when separately incubated with 16S and 23S rRNA isolated from purified ribosomal subunits of both MG1655::ϕPA2 and MG1655::ϕPA8. We found that SAM consumption by the holoenzyme was significantly higher in the presence of 16S rRNA isolated from MG1655::ϕPA2 as compared to 23S rRNA isolated from that strain (Figure 9, left panel black bar, *p*< 0.01). We also found that the methyltransferase activity of the holoenzyme was substantially lower in the presence of either 16S or 23S rRNA isolated from MG1655::ϕPA8 (Figure 9, left white bar, *p*< 0.01). These results indicate that 16S rRNA is a preferred substrate for the holoenzyme.

We observed the identical pattern of methyltransferase activity when the isolated ribosomal subunits were used as substrates. We found that the SAM utilization activity and the degree to which the holoenzyme prefers 16S over 23S rRNA were much higher when these rRNAs were contained within the intact ribosomal subunit (Figure 9, right). This result suggests that the holoenzyme may interact with proteins in the ribosome. Regardless, this result show that the preferred substrate for the holoenzyme is 16S rRNA contained within 30S ribosomal subunit. Taken together, the results in shown here indicate that the M.EcoPA8orf6770P-PNB-2 holoenzyme is a 16S rRNA methyltransferase.

## Discussion

Until recently, lysogenic prophage had been thought to lie dormant within the host genome, and except for carrying pathogenesis-related genes (e.g. antibiotic resistance genes or toxin genes), have little impact on host fitness (82,83). However, it is now known that prophage genomes are not simply passive passengers inserted into the host chromosome, but instead actively regulate host gene expression and consequently control host behavior (50,51,57,84). For example, temperate prophage has been shown to govern host carbon source metabolism, enhance host motility, increase acid resistance and affect intercellular communication (50,54,85,86). Our results presented here extend this paradigm, indicating that a novel phage-encoded RNA methyltransferase regulates induction of Shiga toxin encoding prophage by modifying ribosomal RNA. By doing so, this factor significantly increases the eukaryotic cell killing efficiency of Shiga toxin encoding *E. coli*.

To our knowledge, this is the first report of a prophage-encoded RNA methyltransferase. The absence of identified phage-encoded RNA methyltransferases has been somewhat surprising because as a class, methyltransferases are found ubiquitously, and regulate cellular processes by methylating biopolymers or a vast array of small molecule metabolites (87). Temperate phage, including many Shiga toxin-encoding phage, encode a wide variety of putative DNA methyltransferases, many of which seemingly function to defend the incoming phage against host restriction-modification (R-M) systems (74,88,89). Although it has recently been reported that bacteria do contain mRNA methyltransferases, most known RNA methyltransferase in bacteria modify stable RNAs (e.g, tRNA, rRNA), a feature that is shared by the phage-encoded RNA methyltransferase identified here.

Given this novelty, it is important to acknowledge that the identification of M.EcoPA8orf6770P-PNB-2 holoenzyme as an RNA methyltransferase is based on its pronounced preference for 16S rRNA as the substrate in the SAM utilization assay. We have not yet identified the specific base(s) modified. Regardless, several lines of evidence support the conclusion that M.EcoPA8orf6770P-PNB-2 holoenzyme specifically modifies 16S rRNA. First, we found that the SAM utilization activity of the holoenzyme strongly depends on the type of nucleic acid (RNA vs. DNA). The holoenzyme displays >13-fold higher activity on RNA isolated from bacteria that do not express the methyltransferase as opposed to DNA isolated from the same strain. Second, the activity of the holoenzyme depends on the source of RNA; it has much higher activity with RNA isolated from strains lacking the methyltransferase than from those that express this enzyme. Third, activity of the holoenzyme depends on the type of RNA, showing higher activity in the presence of purified and 30S ribosomal subunit-associated 16S rRNA isolated from methyltransferase-expressing strains, as compared to purified and 50S ribosome-associated 23S rRNA. Fourth, BLAST searches predict that, outside of the bacterial MT-A70 subgroup, the closest sequence relatives to these enzymes contain the IME4-like conserved protein domain. This family is comprised of a number of N6-adenosine-specific RNA methyltransferases and includes METTL14 and METTL3 methyltransferases from mammals, as well other eukaryotic RNA methyltransferases (73). When associated with auxiliary factors, all these proteins specifically transfer methyl groups from SAM to RNA. Moreover, probes of the PDB with the Alphafold-predicted structure (76,77) of M.EcoPA8orf6770P using DALI (90) identified METTL14 and METTL3 methyltransferases as the closest structural homologs of the phage-encoded methyltransferase. This result is also obtained by PHYRE2 threading. Most importantly, we found that the M.EcoPA8orf6770P displays only weak methyltransferase activity in the presence of DNA *in vitro*, including DNA containing unmethylated GATC sequences (Figure 5) is used as a substrate. Taken together, this evidence provides overwhelming support for the conclusion that the phage-encoded M.EcoPA8orf6770P-PNB-2 holoenzyme is primarily a RNA methyltransferase that methylates 16S rRNA.

Interestingly, M.EcoGV, a protein weakly expressed in STEC O104 and highly similar to M. EcoPA8orf6770P, can methylate ^5′^GATC^3’^ sequences in DNA *in vivo* when overproduced from a plasmid (74). However, as seen by Clark et al. (91) and described by Marinus and Løbner-Olesen (92), overproduction of Dam-like methyltransferases can result in off-target methylation *in vivo*. Therefore, Marinus and Løbner-Olesen counsel that caution is warranted when interpreting results from these experiments (92). Additionally, it is also important to note that in the Fang *et al.* (74) study, the plasmid used by to overproduce M.EcoGV did not contain the equivalent of PNB-2, which we demonstrate here is necessary for directing M.EcoPA8orf6770P methyltransferase to target 16S rRNA. As mentioned earlier, full and sequence-specific activity of MT-A70 methyltransferase enzymes usually requires the formation of heteromers with other subunits (75).

Regardless, the observation by Fang, *et al.* (74) suggests that it is possible that M. EcoPA8orf6770P may possess a latent ability to use DNA as a methyltransferase substrate *in vivo*. This idea aligns with our finding that M. EcoPA8orf6770P can, albeit weakly, use DNA as a methyltransferase substrate *in vitro*. Further, this suggestion is also supported by studies showing that METTL3/METTL14-like methyltransferases not only modify N6-adenine in RNA but are also responsible for methylating N6 adenine in DNA of mammals and other eukaryotic cells (93–96). In aggregate, these observations open up the intriguing possibility that M.EcoPA8orf6770P and related methyltransferases may modify both RNA and DNA *in vivo*, a possibility that warrants further exploration.

Current BLAST searches of 607 completely closed STEC genomes on NCBI reveal that genes homologous to that encoding the M.EcoPA8orf6770P rRNA methyltransferase are prevalent in STEC strains, being present in over 34% of these bacteria. Moreover, BLAST analysis of a collection of clinically relevant non-O157 STEC whole genome sequences (97) showed that nearly 40% of these strains contain the DNA sequence that encodes this enzyme. In both cases, the vast majority of these strains (>75%) encode Stx2a. In line with their collaborative function, we discovered that >83% of STEC carrying a gene encoding full-length M.EcoPA8orf6770P (see below) also possess an adjoining gene that encodes PNB-2. In contrast, we found that only about 14% of STEC containing genes that direct expression of proteins identical to M.EcoPA8orf6770P harbor any recognizable PNB-1 coding sequence. Although a large fraction of the genes encoding proteins similar to PNB-1 are located adjacent to that coding for PNB-2, many are not. Although we have not formally ruled out a role for PNB-1 in governing the activity or substrate specificity of M. EcoPA8orf6770P, this observation, along with the finding that the M.EcoPA8orf6770P and PNB-1 are not co-expressed, strongly indicates that PNB-1 does not play a role in the methyltransferase activities of M.EcoPA8orf6770P.

Some non-STEC bacteria encode a truncated isoform of M.EcoPA8orf6770P methyltransferase that lacks the last 27 amino acids of the full-length gene present in STEC. Importantly, no STEC strains encode this variant. Strains carrying this smaller variant also do not seem to contain the gene encoding the putative 16S rRNA binding subunit, PNB-2. These observations suggest that this region of M.EcoPA8orf6770P may be important for its function in STEC and its ability to form the holoenzyme with PNB-2. We note that most of the C-terminal region of the M.EcoPA8orf6770P sequence is poorly modeled both by PHYRE2 and Alphafold (per-residue confidence score in this region is < 50) (76,77). This condition may be the reason why Alphafold failed to obtain high-confidence models of the M.EcoPA8orf6770P-PNB-2 complex.

We are unsure why less than 100% of STEC containing a gene coding for full length M.EcoPA8orf6770P have an adjacent gene directing expression of PNB-2. It is possible this discrepancy is a consequence of problems encountered in assembling bacterial genomes containing multiple prophages (98,99), a condition typical of wild-type STEC (100,101). Phage genomes are mosaics of functionally related genetic modules (82,102) and this modularity complicates completion of the assembly process (103,73). The failure to completely assemble a closed STEC genome may prevent any given deposited sequence from representing the syntenic connection between the genes encoding M.EcoPA8orf6770P and PNB-2. Regardless while we do not know if this issue explains the discrepancy, our analysis shows the vast majority of M.EcoPA8orf6770P expressing STEC strains also encode PNB-2.

Our results indicate that the M.EcoPA8orf6770P-PNB-2 holoenzyme methylates host *E. coli* 16S ribosomal RNA. Ribosomes direct the synthesis of proteins. Since the induction of prophage involves the synthesis of phage protein, Stx and SOS-related proteins, it is likely the holoenzyme regulates induction by affecting translation in some manner. Several residues in 16S rRNA are methylated by host RNA methyltransferases. These modifications affect translation initiation, subunit assembly control and antibiotic resistance (104,105). These three functional categories give us a hint of how M.EcoPA8orf6770P-PNB-2 could regulate prophage induction and consequently, STEC virulence.

Our results show that the preferred substrate for the M.EcoPA8orf6770P-PNB-2 holoenzyme is 16S rRNA contained within 30S intact ribosomal subunit. Similar to the M.EcoPA8orf6770P-PNB-2 holoenzyme, the preferred substrate of three host *E. coli* 16S rRNA methyltransferases, RsmD, RsmH and RsmI, is the 30S subunit rather than free 16S rRNA. The modifications introduced by these host enzymes affect translation initiation rather than ribosome assembly (105–109). These observations suggest that methylation of 16S rRNA by the M.EcoPA8orf6770P-PNB-2 holoenzyme could affect translation/translation initiation rather than ribosome assembly (104,105). Consistent with this idea, the *E. coli* RsmB methyltransferase, which prefers free 16S as substrate over 30S subunit, affects 30S subunit assembly, only indirectly impacting translation (106).

Some methylations of 16S rRNA affect translation by impacting mRNA selection or tRNA binding (106). Hence if the M.EcoPA8orf6770P-PNB-2 affects translation/translation initiation, it might regulate phage induction by changing the affinity of certain mRNAs bind to ribosome, thereby impel the host cell selectively to express phage-induction-related genes. Alternatively, since Stx is a ribotoxin that targets rRNA in sarcin-ricin loop that is located between the large and small ribosomal subunits, methylation catalyzed by the M.EcoPA8orf6770P-PNB-2 holoenzyme may grant Stx resistance to the STEC strain, thereby increasing the number of active ribosomes and enhancing phage-related protein production.

Regardless of its mechanism of action, the work presented here identifies the M.EcoPA8orf6770P-PNB-2 holoenzyme as a phage-encoded rRNA methyltransferase that is commonly found in Stx2a-encoding bacteriophage. Thus, our results show that Stx prophages do not merely carry the toxin gene but use this enzyme to actively modulate host functions that control the release of this virulence factor. This finding suggests that Stx phages take advantage of a heretofore unrecognized methylation-dependent mechanism for regulating its growth and with that, production and release of a cytolethal toxin.

## Supplementary Material

gkad1150_supplemental_file

## Data Availability

The data underlying this article are available in the University at Buffalo Institutional Repository (UBIR), at http://hdl.handle.net/10477/84796.
