# Supplementary material for: A prophage encoded ribosomal RNA methyltransferase regulates the virulence of Shiga-toxin-producing Escherichia coli (STEC)
Source: Nucleic Acids Res. 2023 Dec 12;52(2):856–71. doi: 10.1093/nar/gkad1150 (PMC10810198; doi:10.1093/nar/gkad1150)
Supplement: gkad1150_supplemental_file [file gkad1150_supplemental_file.pdf]

Supplementary Figures

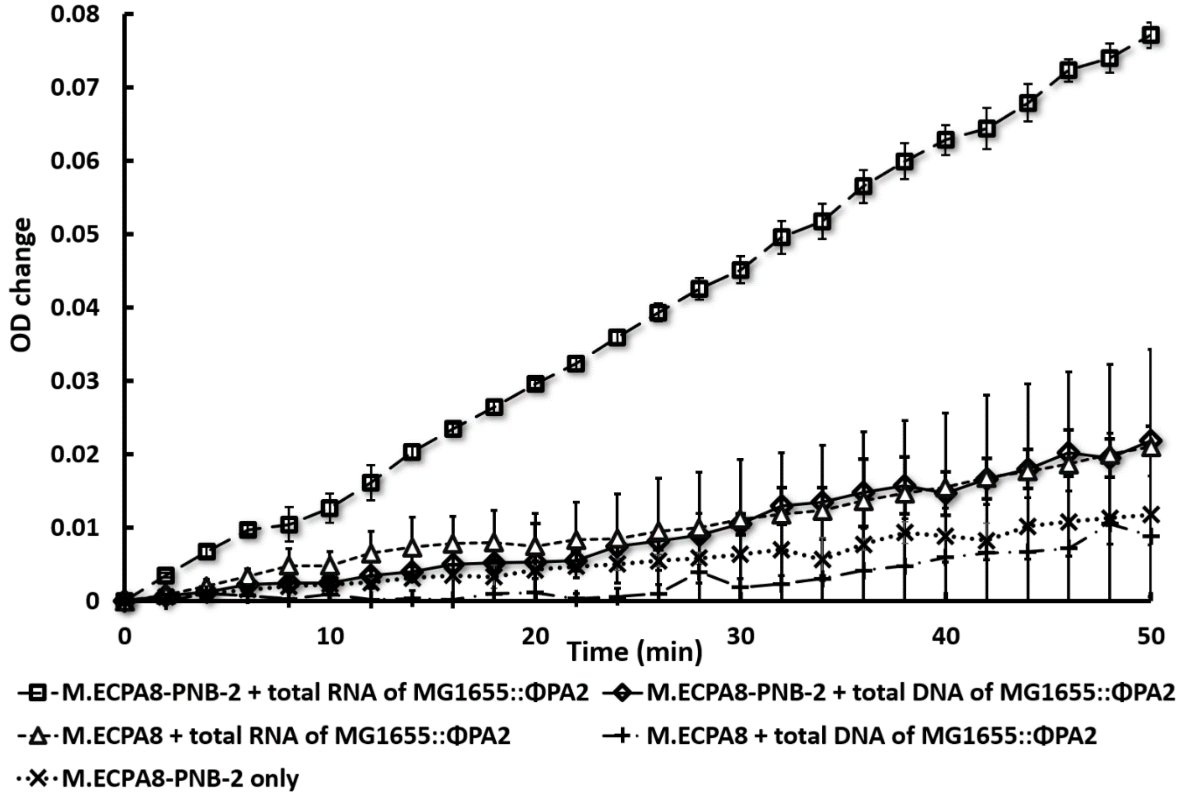

Figure S1. SAM assay curves. Purified M.EcoPA8orf6770P (M.EcoPA8)-PNB-2 holoenzyme or M.EcoPA8orf6770P protein alone was incubated with total RNA or DNA isolated from MG1655::ϕPA2 and the OD change was measured and plot against time as described in Methods and Materials. A background control using the holoenzyme only without any RNA or DNA was also included. Error bars represent standard deviations which were determined from at least 3 biological replicates and each biological replicate was measured from at least 3 technical replicates.

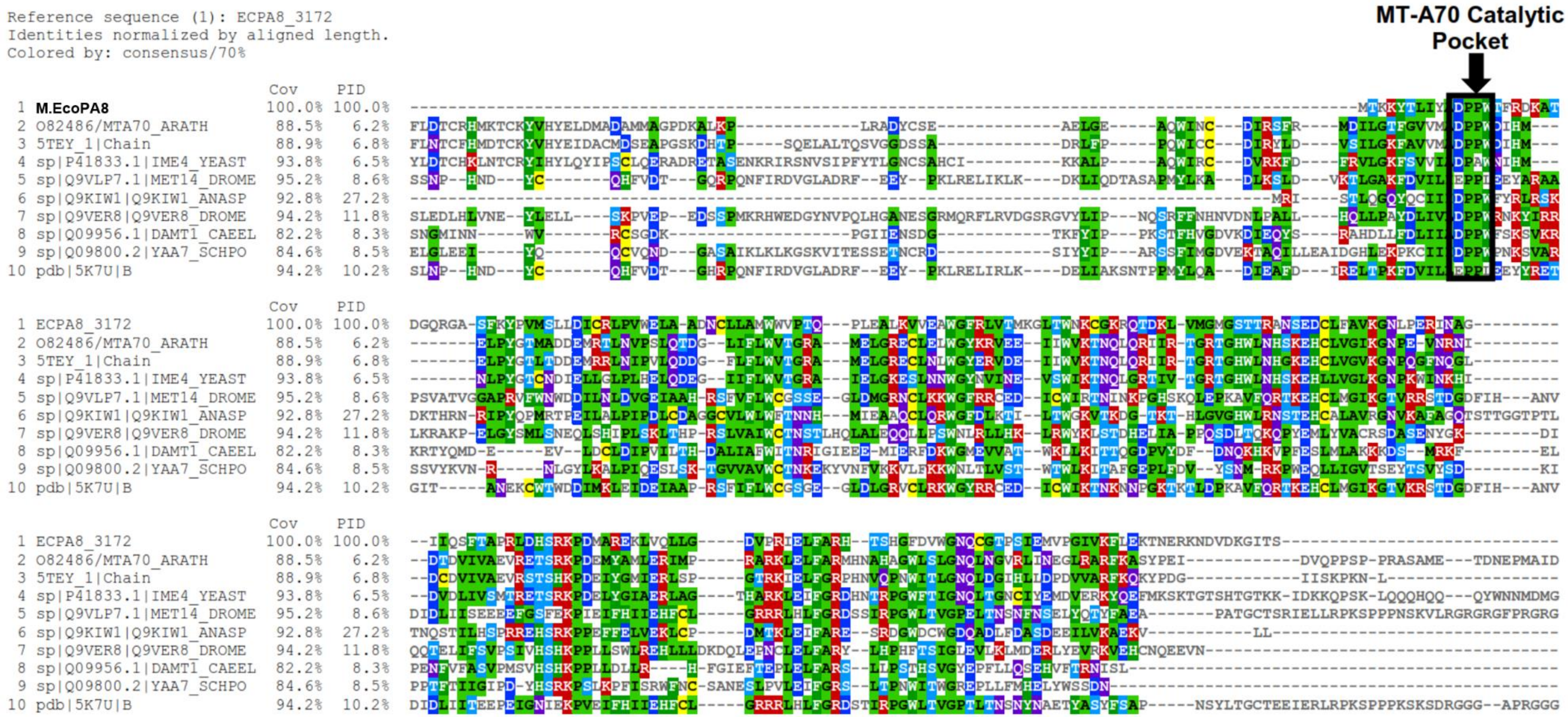
Reference sequence (1): ECPA8_3172
Identities normalized by aligned length.
Colored by: consensus/70%
MT-A70 Catalytic Pocket
Cov PID
1 M.EcoPA8 100.0% 100.0%
2 O82486/MTA70_ARATH 88.5% 6.2%
3 5TEY_1|Chain 88.9% 6.8%
4 sp|P41833.1|IME4_YEAST 93.8% 6.5%
5 sp|Q9VLP7.1|MET14_DROME 95.2% 8.6%
6 sp|Q9KIW1|Q9KIW1_ANASP 92.8% 27.2%
7 sp|Q9VER8|Q9VER8_DROME 94.2% 11.8%
8 sp|Q09956.1|DAMT1_CAEEL 82.2% 8.3%
9 sp|Q09800.2|YAA7_SCHPO 84.6% 8.5%
10 pdb|5K7U|B 94.2% 10.2%
1 ECPA8_3172

Figure S2. The M.EcoPA8orf6770P sequence shares the same conserved DPPW motif with the methyltransferases in MT-A70 superfamily. The figure shows the alignment of M.EcoPA8orf6770P (M.EcoPA8) amino acid sequence against members of the MT-A70 methyltransferases superfamily. The DPPW motif exists in both M.EcoPA8orf6770P and other methyltransferases below. This motif is established as the catalytic pocket of this class of methyltransferases.

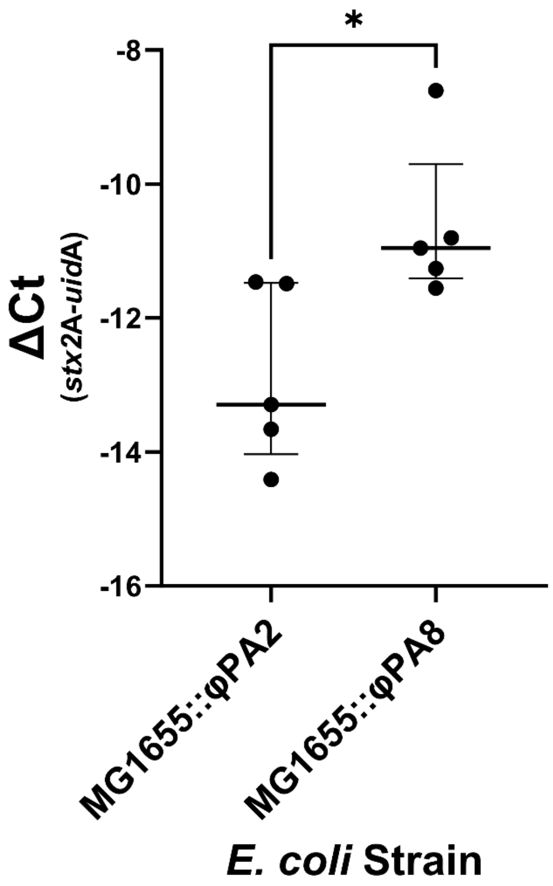

Figure S3. Differential *stx*2A expression in *E. coli* strains containing ϕPA2 or ϕPA8. Total RNA was extracted from MG1655::ϕPA and MG1655::ϕPA8 and cDNA libraries prepared as described in Methods and Materials. These libraries were used in qPCR to measure the amount of *stx2*A transcript relative to the transcript of the housekeeping gene *uid*A. * p <0.03.

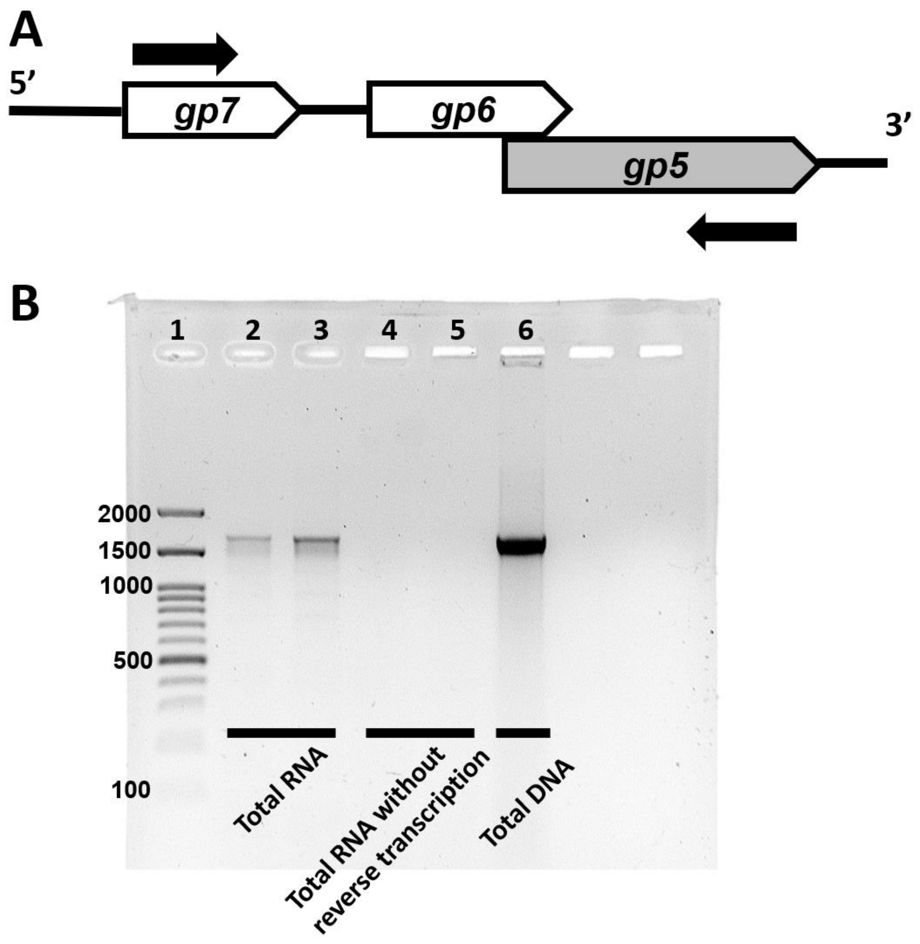

Figure S4. The genes encoding PNB-1, PNB-2 and M.EcoPA8orf6770P are co-transcribed. The total RNA was extracted from the MG1655::ϕPA8 and then was reverse-transcribed into cDNA as described in Methods and Materials. **A.** The cDNA was checked by regular PCR with two primers that amplify the DNA fragment including encoding PNB-1 (*gp*7), PNB-2 (*gp*6) and M.EcoPA8orf6770P (*gp*5) (primers were indicated by the arrows). **B.** The PCR product was displayed on an agarose gel and visualized by ethidium bromide staining. The bands in lane 2&3 represent the cDNA that synthesized from the mRNA molecule which carries all 3 genes on it. The lane 4&5 are PCR reactions using total RNA that has not been subjected to reverse transcription. Lane 6 displays the PCR product obtained using total DNA from MG1655::ϕPA8 as template.

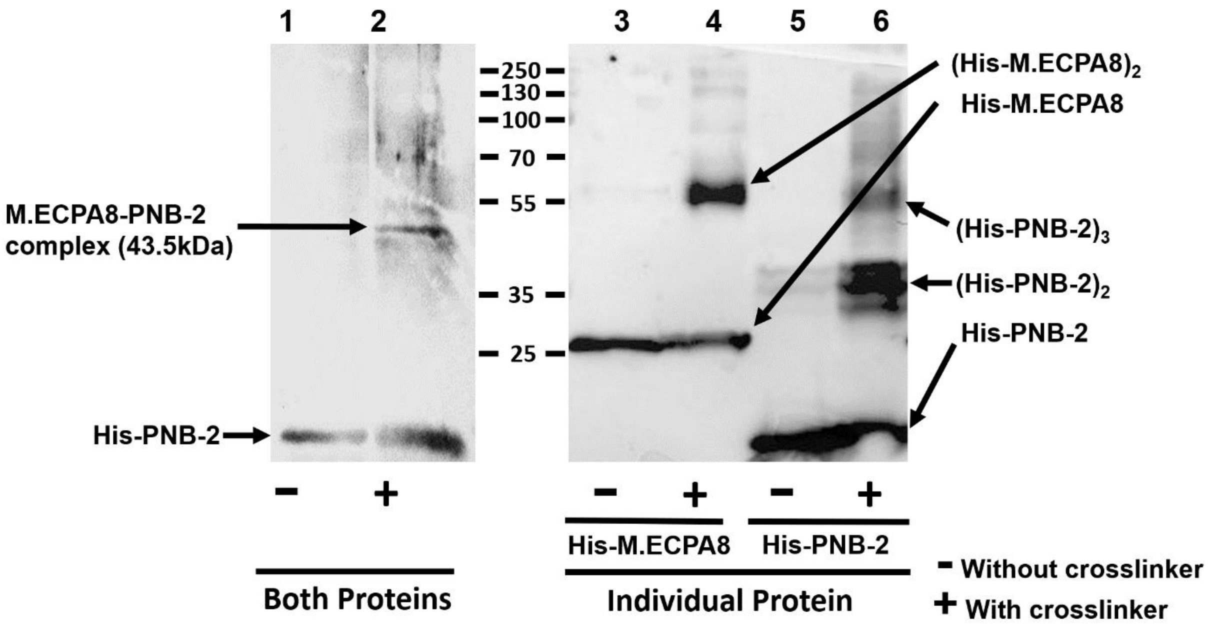

Figure S5. M.EcoPA8orf6770P-PNB-2 holoenzyme contains one of the M.EcoPA8orf6770P protein and one of PNB-2 protein. All products of crosslinking reaction were fractionated on SDS-PAGE gels, electroblotted onto PVDF paper visualized by immunoblot with anti-His-tag antibody as described in Methods and Materials. Lanes 1 and 2 display the products formed when purified M.EcoPA8orf6770P (M.EcoPA8) and His-tagged PNB-2 are incubated in the absence or presence, respectively of dimethyl suberimidate. Reactions containing the individual His-tagged M.EcoPA8orf6770P (lanes 3 & 4) or PNB-2 (lanes 5 & 6) incubated without (lanes 3 & 5) and with (lanes 4 & 6) crosslinker identify the positions of products formed by the individual proteins in presence of crosslinker. The positions of crosslinking products are indicated. The apparent molecular weights of these products do not overlap those of the products formed by crosslinked mixtures of M.EcoPA8orf6770P and His-tagged PNB-2 proteins.
